# Leveraging the Oxford Nanopore MinION sequencing platform for HIV-1 drug resistance surveillance in resource-limited settings: a post-COVID implementation opportunity

**DOI:** 10.1186/s12985-026-03134-0

**Published:** 2026-03-15

**Authors:** Tendai Washaya, Benjamin Chimukangara, Justin Mayini, Sandra Bote, Nyasha Chin’ombe, Shungu Munyati, Justen Manasa

**Affiliations:** 1 Medical Microbiology Unit, Department of Laboratory Diagnostic and Investigative Science, Faculty of Medicine and Health Sciences, University of Zimbabwe, Harare, Zimbabwe; 2 Biomedical Research and Training Institute, Harare, Zimbabwe; 3 Critical Care Medicine Department, NIH Clinical Centre, Bethesda, MD USA; 4 AmpSeq LLC, Gaithersburg, MD USA; 5 AIDS Healthcare Foundation, Harare, Zimbabwe; 6 Faculty of Medicine and Health Sciences, University of Zimbabwe , Harare, Zimbabwe

**Keywords:** HIV-1, Genotyping, Drug resistance, Oxford Nanopore Technologies

## Abstract

**Background:**

HIV drug resistance (HIVDR) testing remains essential for optimizing antiretroviral therapy (ART), yet access remains constrained by high cost, low throughput, and limited availability of Sanger sequencing in resource-limited settings (RLS). Recent improvements in Oxford Nanopore Technologies (ONT) sequencing offer a potential alternative, especially following the expansion of genomic surveillance infrastructure established during the COVID-19 pandemic.

**Methods:**

A cross-sectional laboratory validation study was conducted comparing ONT MinION sequencing with Sanger sequencing for HIV-1 protease/reverse transcriptase (PR/RT) and integrase (IN) genotyping. Sixty-four stored amplicons from patients with virological failure on second-line protease inhibitor (PI)- or integrase strand transfer inhibitor (INSTI)-based regimens were sequenced on the MinION Mk1C (R10.4.1). ONT-derived consensus sequences were generated using a custom pipeline and compared to Sanger reference sequences to assess nucleotide identity and drug resistance mutation (DRM) concordance using the Stanford HIVdb v9.8. We also evaluated the cost and scalability of ONT to determine its feasibility for broader implementation.

**Results:**

ONT MinION sequencing produced high-quality consensus sequences with a median pairwise identity of 99.4% (IQR: 99.2–99.7). After resolving initial discrepancies through chromatogram review and high-depth Nanopore data, high concordance for major DRMs was observed across PR, RT, and IN coding regions. Using a 48-sample multiplexing configuration, the estimated reagent and consumable cost for ONT sequencing was approximately US$23.47 per sample. Published Sanger-based HIV drug resistance assays report per-sample costs in the range of approximately US$43 to US$100, although costs vary depending on laboratory workflow and testing volume. Moreover, ONT provided a substantially shorter turnaround time (~ 5 h from library prep to sequence data), offering a more efficient and cost-effective workflow overall.

**Conclusions:**

ONT MinION sequencing provides an accurate, rapid, and cost-effective alternative to Sanger sequencing for HIVDR genotyping in RLS. Its scalability, ability to detect minority variants, and compatibility with infrastructure setup up in response to the COVID-19 pandemic, make it a viable platform for both national HIVDR surveillance and decentralized clinical testing. Integrating ONT workflows into routine HIVDR monitoring could expand diagnostic access and enable more timely ART optimization in high-burden settings.

**Supplementary Information:**

The online version contains supplementary material available at 10.1186/s12985-026-03134-0.

## Background

The advent of next-generation sequencing (NGS) technologies has fundamentally transformed the landscape of pathogen detection and genomic surveillance. Among these, the Oxford Nanopore Technologies (ONT) MinION sequencer stands out for its portability, scalability, and capacity for real-time analyses. These features make it particularly attractive for use in Resource Limited settings (RLS), where traditional methods such as Sanger sequencing often remain inaccessible due to high costs, low throughput, and long turnaround times [1]. While NGS holds great potential, its widespread implementation in RLS for applications such as HIV-1 drug resistance (HIVDR) testing, have been hindered by infrastructural demands, bioinformatics expertise, and a lack of sustained funding [2]. Sanger sequencing remains the gold standard for HIVDR testing due to its well-established accuracy and reliability. However, its high operational cost, limited throughput, and long turnaround time have restricted its accessibility in many settings.

Notably, the global COVID-19 pandemic highlighted the importance of NGS technologies, particularly in RLS, where the urgent need for genomic surveillance became a public health priority [3]. This led to the deployment of ONT MinION devices and training of local personnel in sequencing workflows within RLS. Consequently, laboratories in RLS now possess the infrastructure and technical expertise required for high-throughput sequencing. Repurposing this capacity for HIVDR surveillance offers a timely and cost-effective strategy to address persistent gaps in HIV care [4].

The MinION platform presents several advantages for HIVDR testing in RLS. Its relatively low cost, combined with the ability to multiplex samples, enables economies of scale that reduce per-sample costs [5]. Its long-read capability provides better genome coverage and simplifies read alignment [6]. Furthermore, the development of open-source, user-friendly bioinformatics pipelines tailored for MinION HIVDR data analysis have improved accessibility, even in laboratories with limited computational resources [7]. Together, these innovations position the MinION as a transformative tool for expanding equitable access to HIVDR surveillance in underserved regions. The persistent threat of antiretroviral resistance, amid the expansion of antiretroviral drug classes, warrants scalable and affordable tools for HIVDR surveillance.

Therefore, this study evaluated the use of MinION sequencing for HIVDR testing in RLS, focusing on technical implementation, comparative performance against conventional Sanger sequencing, and the broader implications for HIV-1 treatment and public health. Leveraging existing infrastructure and capacity, we propose a pragmatic model for integrating NGS into routine HIVDR surveillance and clinical care, aiming to improve outcomes for populations most impacted by the HIV-1 epidemic. We hypothesized that ONT MinION sequencing demonstrates high concordance with Sanger sequencing for HIVDR detection, while providing reduced costs and faster turnaround times suitable for deployment in RLS.

## Materials and methods

### Study design

This was a cross-sectional, laboratory-based validation study designed to evaluate the performance of the ONT MinION sequencing platform for HIVDR testing in comparison to the standard Sanger sequencing method. The analytical concordance between the two platforms in detecting HIVDR mutations and interpreting antiretroviral resistance was assessed. This design was chosen as it provides an optimal framework for evaluating the agreement and accuracy of a novel diagnostic method against an established gold standard.

Stored amplicons of the HIV-1 protease and reverse transcriptase (PR/RT) or integrase (IN) coding regions of the *pol* gene with known HIVDR mutations previously sequenced by Sanger sequencing were obtained for analysis. These amplicons were derived from patients failing protease inhibitor (PI) or integrase strand transfer inhibitor (INSTI)-based second-line regimens at provincial and central hospitals across Zimbabwe, between January 2021 and December 2024, through the Zimbabwe National HIVDR Testing Program. This is a public health initiative designed to expand access to HIVDR testing across public healthcare facilities in Zimbabwe. We obtained demographic and clinical information from patient request forms.

### Genotypic resistance testing with Sanger sequencing and reference data generation

Viral HIV-1 RNA was extracted from 500 µL plasma with a confirmed viral load > 1000 copies/mL using the NucliSens easyMAG system (bioMérieux) and eluted in 25 µL. The extracted RNA (10µL) was amplified using the Applied Biosystems TaqPath Seq HIV-1 Genotyping Assay Kit (ThermoFisher Scientific, Waltham, MA, USA), according to manufacturers’ instructions. The quality of the nested PCR product was assessed on a 1% agarose gel against an O’GeneRuler 1 kb DNA ladder as a molecular weight marker. Successfully amplified samples had sequenced for the PR, RT, and IN coding regions, on a SeqStudio Genetic Analyzer (ThermoFisher Scientific, Waltham, MA, USA) as previously described [8], following standardized protocols for HIV-1 pol gene amplification and sequencing. Sequence assembly and editing were conducted using Geneious Prime software (2024.0.7) [9], and resistance interpretation was performed using the Stanford HIV Drug Resistance Database (Stanford HIVdb) v9.8 [10]. The curated Sanger sequences served as the reference standard against which Nanopore-derived mutation profiles and resistance scores were evaluated.

### Library preparation for MinION sequencing

Library preparation of the PR, RT and IN amplicons was performed using the Rapid Sequencing kit v14 (SQK-RAD114) from ONT. Agencourt AMPure XP beads (Beckman Coulter) were used at a 1:1 bead-to-sample ratio for purification and sample libraires were quantified using the Qubit dsDNA HS Assay kit (Invitrogen),. For barcoding, 50ng of DNA per sample was incubated at 30 °C for 2 min followed by 80 °C for 2 min.

The barcoded amplicons were pooled, followed by a second purification with AMPure XP beads, and DNA quantification, to ensure a yield of approximately 800ng. Adapter ligation was performed by adding 1µL of diluted Rapid Adapter to the pooled barcoded DNA, followed by incubation at room temperature for 5 min, producing final libraries ready for sequencing.

### Nanopore sequencing

The library was sequenced on a MinION Mk1C device using a R10.4.1 Flow Cell (FLO-MIN114) and a platform quality control check was performed to verify at least 800 active nanopores, as recommended by the manufacturer. Sequencing libraries were loaded onto the flow cell and run with MinKNOW software, using a 12-hour sequencing and base calling protocol. Sequencing runs were monitored in real time and manually stopped once sufficient data had been generated, defined as an average read depth of ≥ 1000x, ≥ 90% genome completeness, and a total data yield of approximately 200–250 Mb. For a batch of 48 multiplexed samples, these thresholds would be typically reached after about 5 h run time.

### Sequence data analysis

Following sequencing, FastQ reads generated from the MinION platform were processed using a custom HIVDR analysis pipeline developed in this study, targeting the HIV-1 pol gene regions (PR, RT, and IN). Briefly, sequences were filtered using NanoFilt to remove low-quality reads (Phred score < 7), and the remaining reads were aligned to the corresponding *pol* gene regions of the HXB2 reference genome using Minimap2 v2.24. The resulting alignments were polished with Medaka v1.7.3 to generate high-accuracy consensus sequences for downstream analysis. To ensure uniformity and eliminate software-specific bias, Geneious Prime was used for consensus generation and variant calling for both Sanger and ONT datasets. ONT consensus sequences were not generated from intermediate text files but were derived directly from aligned BAM files imported into Geneious Prime, using the internal SNP caller set to a 3% minimum frequency. The consensus FASTA files were then downloaded and submitted to the Stanford HIVdb for resistance interpretation.

### Comparative analysis and validation metrics

The performance of Nanopore sequencing for HIVDR detection was evaluated by comparing consensus sequences generated by the custom ONT pipeline with previously obtained Sanger sequences from same amplicon samples. The comparison was conducted across three key metrics: sequence identity, mutation concordance, and drug resistance interpretation.

1. *Sequence identity analysis*

Paired consensus sequences from the ONT and Sanger platforms were aligned using ClustalW and visualized in Geneious Prime software [9] using the ‘Match-on-overlap’ identity setting. Under this scoring model, a match is recorded if compared bases share at least one overlapping nucleotide (e.g., an ‘R’ [A/G] is counted as a 100% match to ‘A’, ‘G’, or another ‘R’). This method ensures that shared nucleotides within the viral quasispecies are correctly identified, preventing the artificial inflation of genetic distance due to minor variations in base-calling at mixed sites. Pairwise nucleotide identity (%) was calculated to assess overall sequence similarity, and discordant regions were manually inspected, with particular attention to known error-prone homopolymer regions in Nanopore reads.

2. *HIVDR mutation concordance*

Based on HIVDR mutations identified from both ONT- and Sanger-derived consensus sequences, mutations at key codon positions across the *pol* gene (PR, RT, and IN) were compared, focusing on major resistance-associated mutations, as defined by WHO and IAS-USA guidelines [11] (Supplementary Table S1). A mutation-level concordance table was generated to summarize agreement in HIVDR mutation detection between the two platforms.

### Ethics

This study obtained research ethics approval from the local institutional review board of the University of Zimbabwe Joint Research Ethics Committee. Additional approvals were obtained from the BRTI Ethics Research Board, the Parirenyatwa Group of Hospitals (JREC/362/2021), and the Medical Research Council of Zimbabwe (MRCZ/A/2847).

## Results

### Sequence workflow comparison

**Fig. 1:**
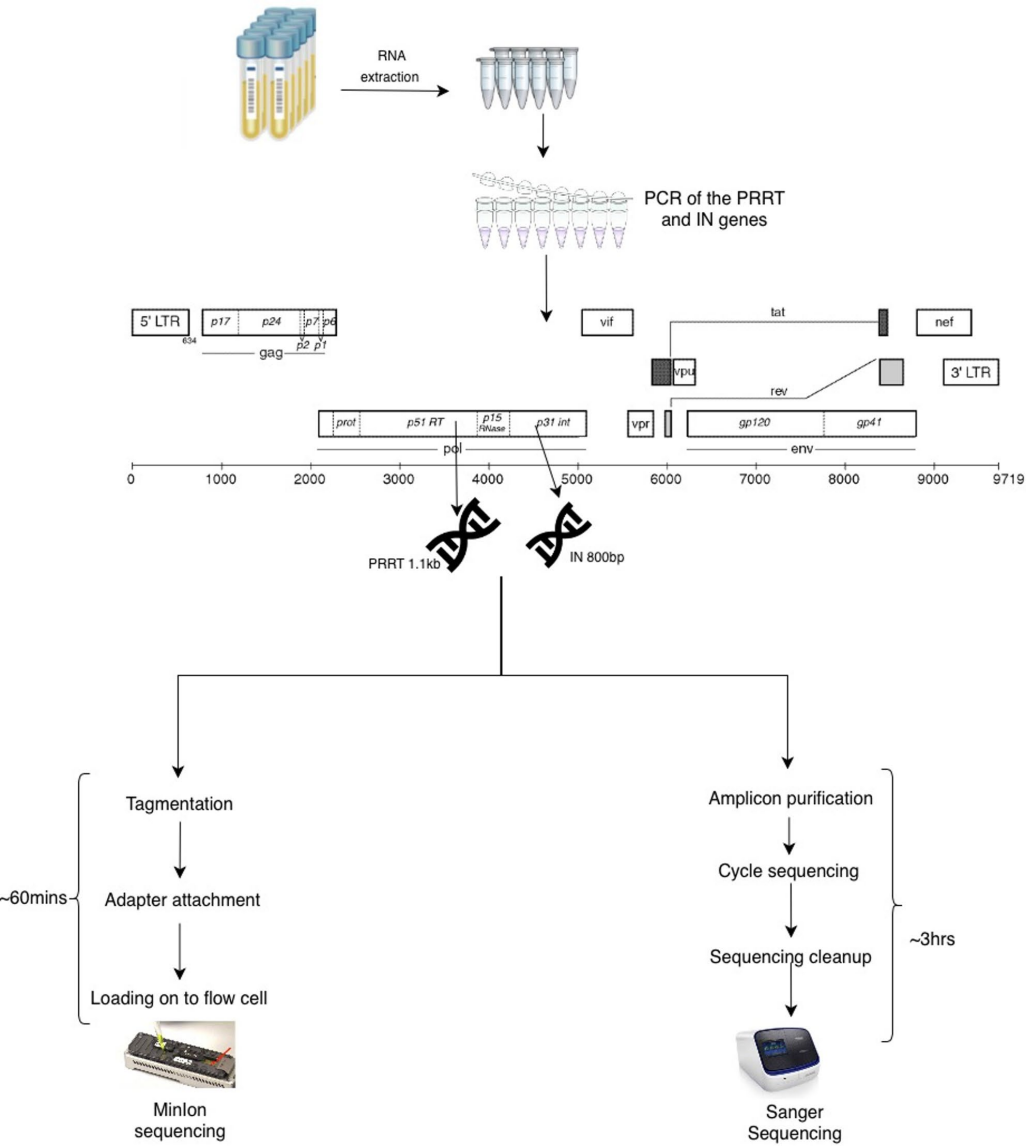
HIV-1 drug resistance sequencing workflow using ONT MinION and Sanger sequencing methods. The HIV-1 genome structure was adapted from the Los Alamos National Laboratory (LANL) HIV Sequence Database [12]. IN, *integrase* gene; PCR, polymerase chain reaction; PR/RT, *protease* and *reverse transcriptase* genes; RNA, ribonucleic acid

A total of 64 amplicons, previously genotyped by Sanger sequencing, were analyzed to compare the laboratory workflows and sequencing performance of the ONT MinION and Sanger platforms for HIVDR testing. Both methods shared a common upstream workflow, involving RNA extraction from plasma samples followed by PCR amplification of the *pol* gene regions, specifically the PR/RT (~ 1.1 kb in size) and IN (~ 800 bp in size) gene fragments (Fig. 1).

Following amplification, the workflows diverged, with the ONT MinION protocol having a rapid library preparation process involving tagmentation, adapter ligation, and direct loading onto the MinION flow cell, requiring approximately 60 min. In contrast, the Sanger workflow involved amplicon purification, cycle sequencing, and post-sequencing processing, extending the turnaround time to about 3 h before data generation (Fig. 1).

### Sequence identity analysis

To ensure accurate alignment, ONT MinION reads were trimmed to match the coverage of the corresponding Sanger sequences, and pairwise identities were calculated across overlapping regions. Comparative analyses between paired ONT MinION and Sanger consensus sequences demonstrated high overall concordance. Across all 64 amplicons, the median pairwise identity was 99.4% (interquartile range (IQR): 99.2–99.7%) (Supplementary Table 1). 89.1% sequences (57/64) showed > 99% identity, confirming the high fidelity of ONT-derived consensus sequences relative to Sanger. All sequences were identified as HIV-1 subtype C.

### Drug resistance concordance

The analysis of major and accessory DRMs included 8 PI major, 12 PI accessory, 14 NRTI, 10 NNRTI, and 8 INSTI (6 major and 2 accessory) amino acid positions. A total of 237 concordant mutation calls ( i.e., amino acid substitutions at drug resistance-associated positions) were identified across all regions. Concordance was also evaluated at all key resistance-associated positions where substitutions were not observed. However, eighteen discordant mutation calls were detected between the ONT Minion and Sanger methods across the PR, RT, and IN regions, as shown in Table 1.

**Table 1:** Comparison of HIV-1 drug resistance mutations detected by Oxford Nanopore Technologies (ONT) and Sanger sequencing

Study ID	PI-major		NRTI mutations		NNRTI		INSTI	
	ONT	Sanger	ONT	Sanger	ONT	Sanger	ONT	Sanger
HIVDR016_RT	I54L *L23I* *G73S* *L89T*	**M46MI** I54L *L23I* ***Q58QE*** *G73S* *L89T*	M184V	**L74LI** M184V	E138A	E138A	NA	NA
HIVDR031_RT	***G73S***		M41L **S68G** **V75VI** M184V	M41L M184V	K103N **V106VM** E138A	K103N E138A	NA	NA
HIVDR033_RT	I50L V82A *L33F* *G73T*	I50L V82A *L33F* ***G73ST***	None	None	None	None	NA	NA
HIVDR215_RT	M46I *K20TL89T*	M46I *K20T L89T*	**D67del** K70R M184V T215I K219E	**D67N** K70R M184V T215I K219E	A98G K103S V108I G190A	A98G K103S V108I G190A	NA	NA
HIVDR254_RT	None	**N88NS**	M184V	M184V	K101E	K101KE	NA	NA
HIVDR272_RT	V32I M46II84VN88S *Q58E* *T74P*	V32I M46I I84V N88S *Q58E* *T74P*	K70E M184V T215Y K219R	**M41ML** K70KE M184V T215Y K219KR	A98G K101E V108I Y181C G190A	A98G K101E V108I Y181C G190GA	NA	NA
HIVDR264_IN	NA	NA	NA	NA	NA	NA	E138K G140A Q148K	E138K G140A Q148K ***G163GR***
HIVDR267_IN	NA	NA	NA	NA	NA	NA	None	***E157EQ***
HIVDR343_RT	V32I I47V *M46V* *F53L* *L89T*	V32I I47V *M46V* *F53L* *L89T*	K65R M184V	K65R **Y115YF** M184V	K101E E138A Y188L G190A	K101E E138A Y188L G190A	NA	NA
HIVDR354_RT	V82L L90M *K20T* *G73T*	V82L L90M *K20T* *G73T*	D67N T69D K70R M184V K219E	D67N **T69DN** K70R M184V K219E	E138A	E138A	NA	NA
HIVDR365_RT	None	None	**K65KN**	None	None	None	NA	NA

PI, protease inhibitors;  INSTI, Integrase strand transfer inhibitors; ONT, Oxford nanopore technologies; NRTI, nucleoside reverse transcriptase inhibitors; NNRTI, non-nucleoside reverse transcriptase inhibitors

*Discordant mutations are highlighted in bold

*Accessory mutations are in italics

To evaluate the comparative diagnostic yield of both platforms, we performed a detailed analysis of all discordant base calls between the Sanger and ONT consensus sequences (Table 2).

**Table 2:** Analysis of discordances between Sanger and ONT MinION

Sample ID	Mutation call (nt sequence)		Analysis of discordances
	ONT	Sanger	
HIVDR016_RT^*^	None (ATG) None (CAG) None (TTA)	M46MI (ATR) Q58QE (SAG) L74LI (WTA)	Sanger had low signal-to-noise ratio ONT showed no mutation at all sites
HIVDR031_RT^*^	Missing Missing Missing Missing	None (GGT) None (AGT) None (AAA) None (GTG)	Sanger sequencing failure due to primer fail; ONT high-depth reads provided full coverage of the region
HIVDR033_RT^*^	G73T (ACT)	G73ST (AST)	Sanger showed high noise to signal from ‘A’ and ‘C’ baseline noise, suggesting a mixture of Serine (AGT) and Threonine (ACT). ONT high depth detected only the threonine (ACT) variant
HIVDR215_RT^*^	D67del (__)	D67N (AAT)	Sanger high noise than signal, ONT high depth identified a deletion at the position
HIVDR254_RT	None (AAY)	N88NS (ARY)	Sanger reported an ambiguous mixture (R/Y); ONT high-depth detected only wild-type
HIVDR272_RT	M41M (ATG)	M41ML (MTG)	Sanger detected a mixture (A/C), ONT high depth analysis showed no statistical support for the minor ‘C’ allele at our 3% threshold
HIVDR264_IN	None (GGG)	G163GR (GGK)	Sanger showed a mixture (G/T); high-depth ONT analysis identified only the glycine (GGG) allele
HIVDR267_IN	None (GAA)	E157EQ (CAR)	Sanger reported an ambiguous C/A mixture (R); high-depth ONT showed a consensus for the glutamate (GAA) allele
HIVDR343_RT	None (TAT)	Y115YF (TWT)	Sanger showed a mixture (A/T); ONT high-depth identified wild-type TAT
HIVDR354_RT	T69D (GAT)	T69DN (RAT)	Sanger reported an ambiguous A/G mixture (R); high-depth ONT provided a clear GAT consensus
HIVDR365_RT	K65KN (AAW)	None	Minority variant detected at 3.5% VAF (x1,479 depth); falls below the established 20% sensitivity limit of Sanger sequencing

nt, nucleotide; IN, Integrase; ONT, Oxford Nanopore technologies; RT, reverse transcriptase

Sample IDs marked with an asterisk (*) represent cases where the Sanger chromatogram exhibited sub-optimal quality (e.g., low signal-to-noise ratio or incomplete primer coverage) according to standard WHO QA criteria. These are reported here to illustrate the gap in diagnostic yield between the two platforms

As noted in Table 2, several Sanger sequences exhibited technical limitations, including low signal-to-noise ratios and incomplete primer coverage that would traditionally render them unreportable under strict quality assurance (QA) frameworks. Rather than excluding these samples, we analyzed them to evaluate the comparative diagnostic yield of the ONT platform. In these instances of Sanger technical failure (e.g., HIVDR016_RT, HIVDR031_RT), high-depth ONT sequencing (> 1,000×) successfully ‘rescued’ the samples, providing full-length, high-confidence consensus profiles where Sanger traces were obscured by baseline noise. Furthermore, discordances in mixture calls (e.g., HIVDR254_RT) were investigated through manual inspection. While Sanger suggested ambiguous mixtures, these often coincided with signal artifacts; the high-depth ONT data provided a clear consensus, identifying these sites as wild-type with high statistical confidence. Additionally, ONT identified minority variants (3.5% VAF in HIVDR365_RT) that fall below the 20% sensitivity threshold of capillary electrophoresis. Collectively, these data indicate that while Sanger remains a gold standard for high-quality amplicons, ONT demonstrated the capacity to generate high-confidence consensus sequences in samples where Sanger chromatograms were suboptimal.

### Cost and scalability analysis

We further assessed the economic viability of implementing the ONT MinION platform for national HIVDR surveillance through a comparative cost analysis with the standard Sanger sequencing method, focusing on capital expenditure and per-sample reagent costs.

### Initial capital investment

The major economic advantage of the ONT MinION platform workflow lies in its minimal upfront capital requirement. In contrast, a dedicated Sanger sequencing instrument entails a substantial initial investment, typically ranging from US$10,000 to over US$200,000 for the used core instrument and necessary computational infrastructure [13], representing a major barrier to adoption in RLS. By comparison, the current-generation Nanopore, exemplified by the MinION Mk1D Pack, is listed at approximately US$4,950.00 [14], including the sequencer, flow cells, and basic control kits. This represents only a fraction of the cost of a dedicated Sanger instrument, effectively eliminating the high capital barrier. Although higher than the entry price of around US$1,000 for earlier ONT MinION models, this remains a significantly lower initial investment for a fully functional sequencing platform.

### Per-sample costing

Beyond the initial capital investment, the Nanopore platform offers a distinctly lower cost per sample, as its recurring expenses depend on the extent of sample multiplexing, making it scalable. With a high-throughput setup and assuming a 48-sample multiplexing batch, the cost of key consumables (i.e., flow cell and barcoding kit) is shared across all samples. This high level of multiplexing reduces the estimated reagent and flow cell cost to approximately $23.47 per sample (Table 3). Such a significant cost reduction, driven by the platform’s efficient scaling of consumable use, is essential for making routine and large-scale HIVDR surveillance economically viable for public health programs. Further cost optimization is possible with ONT sequencing through flow cell washing and reuse, depending on residual pore availability and sequencing output requirements. Additionally, alternative SPRI-based magnetic bead formulations may be substituted for proprietary bead systems to reduce consumable expenses. These factors suggest that the per-sample reagent cost presented here may represent a conservative estimate under routine laboratory conditions.

**Table 3:** Cost estimate for HIV-1 library construction and sequencing using the ONT MinION platform

Reagents/consumables	Cost per 48 samples (USD)	Cost per sample (USD)
AMPure XP beads	$264	$5.50
Rapid barcoding kit 96 v14	$550	$11.46
Qubit dsDNA HS Assay Kit	$1.48	$0.06
MinION Flow cell	$300	$6.25
Microcentrifuge tubes	$0.40	$0.01
Qubit assay tubes	$2.63	$0.06
Filter tips	$6.70	$0.14
Total	$1125.21	$23.47

AMPure XP, Agencourt Pure Extra Performance beads; dsDNA, double-stranded DNA; HS, high sensitivity; USD, United States Dollar

### Work-flow efficiency

In addition to reagent costs, workflow efficiency differed substantially between platforms. The ONT rapid library preparation process required approximately 60 min of hands-on time, followed by automated base calling and consensus generation. In contrast, Sanger sequencing workflows typically involve longer preparation time and manual chromatogram inspection, particularly for the interpretation of mixed-base calls. Although technician labour costs were not formally monetized in this analysis, reduced manual intervention and automated consensus generation may further improve operational efficiency for ONT-based testing, particularly in high-throughput or surveillance settings.

## Discussion

This study demonstrates the ONT MinION sequencing platform as a reliable, scalable, and cost-effective alternative to conventional Sanger sequencing for HIVDR genotyping in RLS. The high concordance in mutation detection across the HIV-1 *pol* gene regions suggests that recent improvements in ONT’s sequencing chemistry and basecalling algorithms have significantly improved read accuracy, addressing previous limitations associated with homopolymer errors [15]. These findings support the feasibility of integrating ONT sequencing into HIVDR surveillance and clinical diagnostics, offering a practical and sustainable approach for RLS, where traditional methods may be less accessible or economically viable.

The high sequence identity observed between ONT- and Sanger-derived consensus sequences (median 99.4%) highlight the analytical reliability of the ONT platform. These findings are consistent with recent studies showing comparable accuracy between ONT and both Illumina and Sanger sequencing for a range of viral pathogens, suggesting that the ONT technology has reached a level of maturity suitable for clinical and surveillance applications [16–18]. Moreover, ONT’s long-read capability enables the resolution of within-host viral diversity and minority variants that may be missed by population-based Sanger sequencing, providing a more comprehensive understanding of the resistance landscape, an advantage particularly relevant in RLS, where patients often harbor complex mutational patterns due to extensive treatment histories [19].

Despite the strong concordance, a limited number of discordant mutations were observed between the platforms. As noted in our analysis of discordances (Table 2), several samples that failed to meet traditional Sanger QA standards due to low signal-to-noise ratios or primer interference were successfully sequenced using ONT. In a public health surveillance context, these samples would traditionally require costly re-amplification or be discarded as unreportable. The ability of high-depth NGS to ‘rescue’ these samples suggests that ONT can significantly increase the diagnostic yield of HIVDR programs, particularly in settings where cold-chain issues or low viral loads may compromise template quality. Furthermore, ONT detected minority variants, such as the 3.5% VAF K65N in HIVDR365, which remained were not detectable by Sanger sequencing. We also acknowledge that the observed discordances between ONT and Sanger sequencing are influenced by stochastic effects and the presence of viral mixtures. These discrepancies likely reflect the inherent limitations of population-based sequencing and technical noise near the detection limit, particularly for variants close to our 3% threshold. While ONT’s high sequencing depth aims to stabilize these calls, stochastic variation during the initial PCR amplification remains a contributing factor for both methods, potentially leading to the differential detection of low-frequency variants. These findings align with prior studies demonstrating that ONT can sensitively detect mixed viral populations and linked resistance mutations not consistently captured by Sanger [4, 20].

When considering the economic feasibility of transitioning to NGS, it is important to note that costs are highly dependent on laboratory-specific variables, including test volume, labor rates, and the specific protocol used. While our ONT-based method showed a reagent cost of $23.47, Sanger-based assays reported in the literature vary widely. For example, some specialized in-house Sanger protocols have achieved costs as low as $43 USD [21], whereas others exceed $100 USD [22].While ONT may offer a lower ‘per-base’ cost and higher throughput, the total cost-effectiveness for a specific national program will depend on the existing infrastructure and the ability to batch samples effectively to maximize flow cell utility.

The scalability of ONT makes it an attractive tool for strengthening national HIVDR surveillance. Multiplexing enables dozens of barcoded samples to be sequenced on a single flow cell, producing larger and more geographically representative datasets at a substantially lower cost than conventional methods [23–25]. Such real-time surveillance enhances the early detection of emerging resistance patterns and supports timely ART guideline adjustments, in line with WHO recommendations for sustainable HIVDR monitoring [1]. Clinically, the rapid availability of genotyping results allows earlier regimen optimization and reduces the risk of prolonged virological failure and onward transmission of resistant variants. ONT’s improved sensitivity for minority variants may further enhance individualized treatment planning, particularly in heavily treated patients with complex resistance profiles [4].

Our findings align with recent 2024 validations of ONT for HIVDR, such as Ode et al. (2024) and Park et al. (2024), which confirm the platform’s high concordance with standard-of-care methods [24, 25]. However, our work provides a critical implementation perspective by focusing on the diagnostic yield gain. In many RLS environments, the ability to successfully generate a reportable sequence from a single PCR attempt is as vital as the accuracy itself. We demonstrate that ONT can ‘rescue’ samples that would traditionally be discarded due to Sanger technical failures or baseline noise. This operational robustness reduces the need for costly re-amplification or sample recollection, offering a practical blueprint for national surveillance programs to achieve sensitive results despite the logistical challenges of localized reagent procurement and variable template quality.

Ongoing advancements in ONT technology and bioinformatics are anticipated to strengthen its utility for HIVDR testing further. Improvements in flow cell chemistry, adaptive sampling, and real-time variant calling are projected to increase both accuracy and throughput [7]. Meanwhile, the introduction of locally deployable analysis tools could make ONT sequencing more practical in regions with limited internet connectivity [15]. Moreover, integrating ONT-based workflows into existing COVID-19 genomic surveillance systems offers a unique opportunity to expand laboratory capacity with minimal additional investment, thereby strengthening pathogen genomic monitoring across Africa.

To enable broader adoption of MinION-based HIVDR testing, the establishment of supportive policies will be essential. For instance, national programs should consider incorporating ONT sequencing into routine surveillance frameworks and establishing regional sequencing hubs. Sustained investment in digital infrastructure and data management systems will also be necessary to ensure secure and efficient automated analysis pipelines. Furthermore, international partners and donors can play a crucial role by subsidizing consumables, supporting training initiatives, and promoting technology transfer partnerships to ensure equitable access across RLS.

These findings should be interpreted in light of the following limitations. First, sequencing was performed on a relatively small sample size, although adequate for analytical comparison, larger studies in the same context are needed to further validate the study. Second, minority variant detection was not systematically optimized, so the sensitivity of ONT for low-frequency HIVDR mutations requires further evaluation. Thirdly, the lack of Unique Molecular Identifiers (UMIs) to track individual templates. At lower viral loads, high read depth can reflect technical ‘resampling’ of PCR duplicates rather than true biological diversity. In this study, all samples maintained a VL > 1,000 copies/mL (Supplementary Table 2), providing a sufficient template base for detection at our 3% threshold. The high sequence identity observed between Sanger and ONT across the VL spectrum suggests that template bottlenecks did not significantly distort our findings though UMI-based strategies remain the gold standard for absolute quantification of minority variants.

Finally, although the study demonstrates strong analytical concordance under controlled laboratory conditions, broader implementation in decentralized settings will require further operational assessment. Despite these constraints, the findings provide robust evidence supporting the feasibility and accuracy of ONT for HIVDR genotyping in resource-limited settings.

## Conclusion

ONT MinION sequencing provides an accurate, rapid, and affordable alternative to Sanger sequencing for HIVDR testing, with clear advantages for decentralized laboratory networks in RLS. Its strong analytical performance, operational efficiency, and ability to resolve minority variants support its use for both clinical care and national HIVDR surveillance. With appropriate governance, investment, and policy support, ONT has the potential to substantially strengthen HIVDR monitoring and improve treatment outcomes in high-burden, resource-limited settings.

## Supplementary Information

Below is the link to the electronic supplementary material.

Supplementary Material 1

## Data Availability

Sequence accession numbers will be included once published by GenBank.

## Abbreviations

HIV: Human immunodeficiency virus
ART: Anti-retroviral therapy
RLS: Resource-limited settings
HIVDR: Human immunodeficiency virus drug resistance
NRTI: Nucleotide reverse transcriptase
NNRTI: Non-nucleotide reverse transcriptase
PI: Protease inhibitors
INSTI: Integrase strand transfer inhibitors
PR: Protease
RT: Reverse transcriptase
IN: Integrase
WHO: World health organization
