# Supplementary material for: Leveraging the Oxford Nanopore MinION sequencing platform for HIV-1 drug resistance surveillance in resource-limited settings: a post-COVID implementation opportunity
Source: Virol J. 2026 Mar 15;23:107. doi: 10.1186/s12985-026-03134-0 (PMC13104186; doi:10.1186/s12985-026-03134-0)
Supplement: Supplementary file 1 — Supplementary Material 1 [file 12985_2026_3134_MOESM1_ESM.docx]

**Supplementary Table 1** Pairwise sequence identity between Oxford Nanopore (ONT) and Sanger consensus sequences across sequenced amplicons.

| Sequence Name | Pairwise identity | Identical sites | Total length |
| --- | --- | --- | --- |
| HIVDR016_RT | 99.2 | 1019 | 1035 |
| HIVDR019_IN | 99.4 | 862 | 864 |
| HIVDR023_RT | 99.95 | 1034 | 1035 |
| HIVDR027_RT | 99.1 | 1020 | 1035 |
| HIVDR028_RT | 100 | 1034 | 1034 |
| HIVDR029_RT | 99.4 | 1024 | 1035 |
| HIVDR031_RT | 99 | 1017 | 1035 |
| HIVDR032_RT | 99.2 | 1021 | 1035 |
| HIVDR033_RT | 99.2 | 951 | 959 |
| HIVDR033_IN | 99.6 | 784 | 789 |
| HIVDR038_RT | 99.6 | 1030 | 1035 |
| HIVDR041_RT | 98.6 | 1018 | 1035 |
| HIVDR042_RT | 99.6 | 1055 | 1061 |
| HIVDR044_IN | 99.2 | 782 | 789 |
| HIVDR050_IN | 99.9 | 788 | 789 |
| HIVDR215_RT | 99.1 | 1014 | 1024 |
| HIVDR253_RT | 99.2 | 977 | 987 |
| HIVDR253_IN | 99.5 | 781 | 789 |
| HIVDR254_RT | 99.3 | 1028 | 1035 |
| HIVDR254_IN | 98.8 | 209 | 418 |
| HIVDR260_RT | 99.7 | 982 | 987 |
| HIVDR260_IN | 98.6 | 771 | 789 |
| HIVDR264_RT | 99.9 | 986 | 987 |
| HIVDR264_IN | 99.2 | 783 | 789 |
| HIVDR265_RT | 99.2 | 978 | 987 |
| HIVDR265_IN | 99.2 | 779 | 789 |
| HIVDR267_RT | 98.9 | 973 | 987 |
| HIVDR267_IN | 99.3 | 781 | 789 |
| HIVDR272_RT | 99.2 | 1019 | 1035 |
| HIVDR272_IN | 99.2 | 852 | 864 |
| HIVDR273_IN | 99.9 | 789 | 789 |
| HIVDR282_IN | 99.7 | 785 | 789 |
| HIVDR290_IN | 99.2 | 779 | 789 |
| HIVDR290_RT | 99.3 | 978 | 986 |
| HIVDR292_RT | 99.5 | 936 | 989 |
| HIVDR292_IN | 99 | 848 | 869 |
| HIVDR310_IN | 99.6 | 783 | 789 |
| HIVDR310_RT | 98.8 | 948 | 974 |
| HIVDR325_IN | 99.6 | 787 | 789 |
| HIVDR325_RT | 99.4 | 980 | 987 |
| HIVDR326_IN | 99.9 | 788 | 789 |
| HIVDR326_RT | 99.9 | 987 | 987 |
| HIVDR333_IN | 99.7 | 788 | 789 |
| HIVDR339_IN | 98.5 | 774 | 790 |
| HIVDR340_IN | 99.3 | 785 | 789 |
| HIVDR340_RT | 99.9 | 987 | 987 |
| HIVDR343_RT | 99.5 | 1026 | 1035 |
| HIVDR344_RT | 99.95 | 1034 | 1035 |
| HIVDR349_RT | 99.8 | 1032 | 1035 |
| HIVDR350_RT | 99.4 | 1027 | 1035 |
| HIVDR351_RT | 98.9 | 1022 | 1035 |
| HIVDR352_IN | 99.7 | 785 | 789 |
| HIVDR352_RT | 99.5 | 982 | 987 |
| HIVDR353_RT | 99.7 | 1029 | 1034 |
| HIVDR354_RT | 98.7 | 1013 | 1035 |
| HIVDR356_RT | 99.4 | 1028 | 1035 |
| HIVDR359_RT | 99.6 | 1027 | 1035 |
| HIVDR361_RT | 99.7 | 1031 | 1035 |
| HIVDR365_RT | 99.8 | 1033 | 1035 |
| HIVDR366_IN | 99.8 | 786 | 789 |
| HIVDR368_RT | 99.5 | 983 | 987 |
| HIVDR386_IN | 100 | 789 | 789 |
| HIVDR392_IN | 99.4 | 782 | 789 |

**Supplementary Figure S1** Representative image of a mutation discordance that was resolved between Sanger and ONT

**Supplementary Table 2:** Viral loads of samples included in the study.

| Study ID | Viral Load cp/mL | Log 10 VL cp/mL |
| --- | --- | --- |
| HIVDR016_RT | 110000 | 5.04 |
| HIVDR019_IN | 6164 | 3.79 |
| HIVDR023_RT | 1418 | 3.15 |
| HIVDR027_RT | 442346 | 5.65 |
| HIVDR028_RT | 1240 | 3.09 |
| HIVDR029_RT | 5203 | 3.72 |
| HIVDR031_RT | 151000 | 5.18 |
| HIVDR032_RT | 97500 | 4.99 |
| HIVDR033_RT | 23900 | 4.38 |
| HIVDR033_IN | 23900 | 4.38 |
| HIVDR038_RT | 10600 | 4.03 |
| HIVDR041_RT | 3524 | 3.55 |
| HIVDR042_RT | 15000 | 4.18 |
| HIVDR044_IN | 124336 | 5.09 |
| HIVDR050_IN | 296036 | 5.47 |
| HIVDR215_RT | 3838 | 3.58 |
| HIVDR253_RT | 26538 | 4.42 |
| HIVDR253_IN | 26538 | 4.42 |
| HIVDR254_RT | 70000 | 4.85 |
| HIVDR254_IN | 70000 | 4.85 |
| HIVDR260_RT | 8263 | 3.92 |
| HIVDR260_IN | 8263 | 3.92 |
| HIVDR264_RT | 195000 | 5.29 |
| HIVDR264_IN | 195000 | 5.29 |
| HIVDR265_RT | 53487 | 4.73 |
| HIVDR265_IN | 53487 | 4.73 |
| HIVDR267_RT | 26300 | 4.42 |
| HIVDR267_IN | 26300 | 4.42 |
| HIVDR272_RT | 225000 | 5.35 |
| HIVDR272_IN | 225000 | 5.35 |
| HIVDR273_IN | 24600 | 4.39 |
| HIVDR282_IN | 614000 | 5.79 |
| HIVDR290_IN | 45100 | 4.65 |
| HIVDR290_RT | 45100 | 4.65 |
| HIVDR292_RT | 167000 | 5.22 |
| HIVDR292_IN | 167000 | 5.22 |
| HIVDR310_IN | 12382 | 4.09 |
| HIVDR310_RT | 12382 | 4.09 |
| HIVDR325_IN | 403146 | 5.61 |
| HIVDR325_RT | 403146 | 5.61 |
| HIVDR326_IN | 17300 | 4.24 |
| HIVDR326_RT | 17300 | 4.24 |
| HIVDR333_IN | 7000 | 3.85 |
| HIVDR339_IN | 173000 | 5.24 |
| HIVDR340_IN | 9600000 | 6.98 |
| HIVDR340_RT | 9600000 | 6.98 |
| HIVDR343_RT | 7976 | 3.90 |
| HIVDR344_RT | 2354 | 3.37 |
| HIVDR349_RT | 10100 | 4.00 |
| HIVDR350_RT | 126273 | 5.10 |
| HIVDR351_RT | 491000 | 5.69 |
| HIVDR352_IN | 199000 | 5.30 |
| HIVDR352_RT | 2480000 | 6.39 |
| HIVDR353_RT | 136000 | 5.13 |
| HIVDR354_RT | 1938 | 3.29 |
| HIVDR356_RT | 3984 | 3.60 |
| HIVDR359_RT | 20730 | 4.32 |
| HIVDR361_RT | 5114 | 3.71 |
| HIVDR365_RT | 73370 | 4.87 |
| HIVDR366_IN | 8590 | 3.93 |
| HIVDR368_RT | 2640 | 3.42 |
| HIVDR386_IN | 2354 | 3.37 |
| HIVDR392_IN | 3340 | 3.52 |
